# Plasma folate, but not homocysteine, is associated with Apolipoprotein A1 levels in a non-fortified population

**DOI:** 10.1186/1476-511X-12-74

**Published:** 2013-05-22

**Authors:** Elisabet Söderström, Mats Eliasson, Owe Johnson, Göran Hallmans, Lars Weinehall, Jan-Håkan Jansson, Johan Hultdin

**Affiliations:** 1 Department of Medical Biosciences, Clinical Chemistry, Umeå University, 901 85, Umeå, Sweden; 2 Department of Clinical Chemistry, Sunderby Hospital, Luleå, Sweden; 3 Department of Public Health and Clinical Medicine, Medicine, Umeå University, Umeå, Sweden; 4 Department of Medicine, Sunderby Hospital, Luleå, Sweden; 5 Department of Public Health and Clinical Medicine, Nutritional Research, Umeå University, Umeå, Sweden; 6 Department of Public Health and Clinical Medicine, Epidemiology and Global Health, Umeå University, Umeå, Sweden; 7 Department of Medicine-Geriatric, Skellefteå County Hospital, Skellefteå, Sweden

**Keywords:** Apolipoprotein, Homocysteine, Myocardial infarction, Folate, Epidemiology

## Abstract

**Background:**

Elevated total plasma homocysteine (tHcy) in humans is associated with cardiovascular disease but prevention trials have failed to confirm causality. Reported reasons for this association have been that homocysteine and its major genetic determinant methylenetetrahydrofolate reductase (MTHFR) may have an effect on HDL and Apolipoprotein (Apo) A1 levels. We wanted to study if tHcy and its major determinants were correlated with Apo A1 levels in a large population without folate fortification.

**Methods:**

This study was a prospective incident nested case-referent study within the Northern Sweden Health and Disease Study Cohort (NSHDSC), including 545 cases with first myocardial infarction and 1054 matched referents, median age at inclusion was 59 years. Univariate and multiple regression analyzes was used to study the associations between apolipoproteins Apo A1 and B, tHcy, folate and vitamin B12 in plasma as well as MTHFR polymorphisms 677C>T and 1298A>C.

**Results:**

Apo A1 and Apo B were strongly associated with the risk of a first myocardial infarction. tHcy was not associated with Apo A1 levels. Instead, folate had an independent positive association with Apo A1 levels in univariate and multiple regression models. The associations were seen in all men and women, among referents but not among cases. MTHFR polymorphisms had no clear effect on Apo A1 levels.

**Conclusions:**

Analyzing over 1500 subjects we found an independent positive association between plasma folate (major dietary determinant of tHcy) and Apo A1 levels among those who later did not develop a first myocardial infarction. No association was seen between tHcy and Apo A1.

## Background

Hyperhomocysteinemia has been claimed to be a risk factor for cardiovascular disease. Evidence for this has come from meta-analysis based on several epidemiological studies and also from prospective studies [1-7]. The results of randomized trials with homocysteine lowering treatment have failed to show that homocysteine is a causal risk factor [8-10]. This raises the possibility that homocysteine levels could be associated with another casual risk factor.

Several different mechanisms how hyperhomocysteinemia can lead to atherosclerosis are described, such as inflammation, oxidative stress and enhanced lipogenesis [11,12]. Apolipoprotein A1 (Apo A1) is the major protein component of HDL and is believed to have an atheroprotective role through several mechanisms; one of them is reverse cholesterol transport [13-15]. In epidemiological studies elevated tHcy has been negatively associated with high density lipoprotein (HDL) cholesterol [16-21], and more recently with Apo A1 [20,22-25]. The methylenetetrahydrofolate (MTHFR) polymorphism which has a major impact on tHcy levels [26] has also been reported to have an influence on Apo A1 levels [21,27]. Cell studies have shown that mRNA levels of Apo A1 are affected by homocysteine and MTHFR-polymorphism [27]. This would suggest that hyperhomocysteinemia may be associated with an increased risk of atherosclerosis due to decreased expression of Apo A1 [16,23,27-29]. Some studies have failed to demonstrate an association between Apo A1 and / or HDL with tHcy [30-33] and MTHFR polymorphisms [22,31].

Only a few reports have been published regarding the relationship between folate and Apo A1. In a Norwegian case–control study with 107 patients with acute myocardial infarction (MI), whole blood folate correlated positively and tHcy negatively with Apo A1 and HDL [34]. In a larger study in men, no correlation with folate was seen for Apo A1 or HDL [31]. A small quasi-experimental study including 32 patients with type 2 diabetes, where the patients had a tomato intake (200 g per day), which is a rich source of folate, showed after 8 weeks a significant increase of Apo A1 although tHcy did not change [35].

The aim of this study was to investigate if tHcy and its major determinants folate, vitamin B12 and MTHFR polymorphism are correlated with Apo A1 levels in over 1500 subjects, without folate fortification, including both men and women and to see if this correlation was present among those who did and did not develop a first myocardial infarction. Using multiple regression models, an independent positive association was seen between plasma folate and Apo A1 levels among those who later did not develop a first myocardial infarction.

## Results

The baseline characteristics including the apolipoprotein levels and the MTHFR 677 C>T and 1298 A>C distribution, are presented in Table 1. The plasma concentrations of total homocysteine and Apo B were significantly higher and the Apo A1 concentrations lower, in cases compared to referents. The MTHFR genotypes for both cases and referents were in Hardy-Weinberg equilibrium. ORs for the quartiles of apolipoproteins and Apo B/Apo A1 ratio are shown in Table 2. Apo A1 showed a negative association with the risk of a first MI among men. Among women Apo A1 did not show significant relations in the adjusted models. After adjusting Apo A1 with folate levels the findings remained significant. For Apo B positive associations were seen in all models. Apo B/Apo A1 ratio had a positive association both among men and women in the unadjusted model, though stronger among women. After adjustment (model including diabetes) OR increased among women but the opposite effect were seen among men.

**Table 1:** Baseline characteristics of myocardial infarction cases and referents

	**Men**					**Women**				
	**All**	**Cases**		**Referents**		**All**	**Cases**		**Referents**	
		**N**		**N**			**N**		**N**	
Age at inclusion, years	53.4 ± 7.7	387	53.6 ± 7.7^a^	752	53.4 ± 7.7	58.2 ± 7.5	158	58.3 ± 7.5^a^	302	58.1 ± 7.5
Lag time to MI, years	n.a.	387	4.3 ± 2.5	n.a.	n.a.	n.a.	158	3.4 ± 2.4	n.a.	n.a.
BMI, kg/m^2^	26.3 ± 3.6	375	27.1 ± 3.7^b^	727	25.9 ± 3.4	26.3 ± 4.7	86	26.7 ± 4.7	170	26.1 ± 4.8
Diabetes^c^	7.2	361	11.1^d^	695	5.2	7.7	78	9.0	156	7.1
Hypertension^c^	50.5	373	62.2^b^	721	44.4	57.4	84	72.6^d^	165	49.7
Current smoking^c^	24.9	365	38.4^b^	723	18.1	28.3	152	46.7^b^	297	18.9
Alcohol intake, g/day	5.5 ± 5.1	170	4.6 ± 4.6^d^	367	5.9 ± 5.3	2.41 ± 2.70	35	2.4 ± 2.4	78	2.4 ± 2.8
Apo A1, g/L	1.40 ± 0.21	381	1.35 ± 0.21^b^	734	1.42 ± 0.21	1.57 ± 0.26	155	1.51 ± 0.23^b^	295	1.60 ± 0.27
Apo B, g/L	1.21 ± 0.27	381	1.29 ± 0.28^b^	734	1.17 ± 0.26	1.22 ± 0.29	155	1.29 ± 0.26^b^	295	1.18 ± 0.30
Apo B/ Apo A1 ratio	0.89 ± 0.25	381	0.98 ± 0.26^b^	734	0.84 ± 0.23	0.80 ± 0.23	155	0.87 ± 0.2^b^	295	0.75 ± 0.22
Homocysteine, μmol/L	11.41 ± 4.60	370	11.87 ± 3.83^e^	700	11.16 ± 4.94	10.74 ± 4.36	155	12.19 ± 6.21^b^	293	9.98 ± 2.67
Folate, nmol/L	9.69 ± 4.82	369	8.83 ± 4.45^b^	700	10.14 ± 4.94	11.77 ± 10.14	155	10.64 ± 7.70	293	12.37 ± 11.19
B12, pmol/L	331 ± 158	370	329 ± 148	700	332 ± 162	359 ± 207	155	359 ± 260	293	359 ± 173
*MTHFR 677C>T*	n.a.	366	n.a.	693	n.a.	n.a.	148	n.a.	278	n.a.
*CC*	50.8	185	50.5	353	50.9	50.7	80	54.1	136	48.9
*CT*	40.6	150	41.0	280	40.4	39.2	55	37.2	112	40.3
*TT*	8.6	31	8.5	60	8.7	10.1	13	8.8	30	10.8
*MTHFR 1298A>C*	n.a.	367	n.a.	692	n.a.	n.a.	150	n.a.	278	n.a.
*AA*	39.0	136	37.1	277	40.0	43.9	62	41.3	126	45.3
*AC*	48.3	189	51.5	323	46.7	42.8	66	44.0	117	42.1
*CC*	12.7	42	11.4	92	13.3	13.3	22	14.7	35	12.6

Cases and referents were compared by T-test for continues variables and by Chi-square test for independence for categorical variables. Significant p-values are shown in superscript letters: ^a^matched; ^b^p<0.001; ^c^For definition see method section; ^d^p<0.01; ^e^p<0.05; n.a. = not applicable.

Presented as mean ± SD for continuous variables, and as proportions/percent for non-continuous variables.

**Table 2:** Risk for incident myocardial infarction and p for trend for apolipoprotein quartiles (Q1-Q4)

	**N**	**Q1**^**a**^	**Q2**^**a**^	**Q3**^**a**^	**Q4**^**a**^	**P**_**trend**_
Apo A1						
*All subjects*	1555	1.0	0.74 (0.55-0.98)	0.47 (0.35-0.65)	0.41 (0.30-0.57)	<0.001
*Adjusted*^b^	1150	1.0	0.79 (0.55-1.13)	0.66 (0.45-0.97)	0.52 (0.35-0.79)	0.001
*Adjusted*^c^	1077	1.0	0.77 (0.53-1.11)	0.68 (0.45-1.02)	0.50 (0.33-0.76)	0.001
*Men*	1105	1.0	0.73 (0.52-1.02)	0.53 (0.36-0.76)	0.42 (0.28-0.61)	<0.001
*Adjusted*^b^	950	1.0	0.74 (0.50-1.11)	0.63 (0.41-0.97)	0.50 (0.32-0.79)	0.002
*Adjusted*^c^	894	1.0	0.71 (0.47-1.07)	0.62 (0.40-0.97)	0.47 (0.30-0.76)	0.002
*Women*	450	1.0	0.75 (0.44-1.29)	0.36 (0.20-0.67)	0.40 (0.22-0.72)	<0.001
*Adjusted*^b^	200	1.0	0.81 (0.35-1.90)	0.89 (0.35-2.30)	0.62 (0.22-1.74)	0.425
*Adjusted*^c^	183	1.0	0.83 (0.34-2.05)	1.15 (0.41-3.23)	0.61(0.21-1.78)	0.509
Apo B						
*All subjects*	1555	1.0	1.76 (1.25-2.49)	1.69 (1.19-2.41)	3.55 (2.55-4.95)	<0.001
*Adjusted*^b^	1150	1.0	1.58 (1.02-2.43)	1.75 (1.12-2.74)	3.11 (2.05-4.73)	<0.001
*Adjusted*^c^	1077	1.0	1.78 (1.13-2.81)	1.65 (1.03-2.65)	3.56 (2.29-5.54)	<0.001
*Men*	1105	1.0	1.54 (1.03-2.30)	1.43 (0.93-2.20)	3.34 (2.29-4.87)	<0.001
*Adjusted*^b^	950	1.0	1.45 (0.91-2.30)	1.46 (0.89-2.40)	2.91 (1.86-4.55)	<0.001
*Adjusted*^c^	894	1.0	1.57 (0.97-2.54)	1.35 (0.80-2.26)	3.21 (2.00-5.15)	<0.001
*Women*	450	1.0	2.50 (1.27-4.92)	2.44 (1.27-4.68)	4.13 (2.07-8.25)	<0.001
*Adjusted*^b^	200	1.0	2.62 (0.71-9.65)	3.82 (1.07-13.60)	3.87 (1.08-13.91)	0.036
*Adjusted*^c^	183	1.0	3.86 (0.89-16.81)	4.56 (1.09-19.20)	5.53 (1.32-23.11)	0.024
Apo B/Apo A1						
*All subjects*	1555	1.0	1.48 (1.01-2.16)	2.12 (1.47-3.06)	4.51 (3.19-6.38)	<0.001
*Adjusted*^b^	1150	1.0	1.25 (0.79-1.96)	1.95 (1.24-3.06)	3.06 (2.01-4.66)	<0.001
*Adjusted*^c^	1077	1.0	1.39 (0.86-2.23)	2.17 (1.35-3.50)	3.46 (2.22-5.40)	<0.001
*Men*	1105	1.0	1.33 (0.85-2.07)	1.84 (1.20-2.82)	4.14 (2.78-6.16)	<0.001
*Adjusted*^b^	950	1.0	1.18 (0.72-1.92)	1.71 (1.05-2.76)	2.92 (1.85-4.60)	<0.001
*Adjusted*^c^	894	1.0	1.24 (0.75-2.07)	1.86 (1.12-3.09)	3.24 (2.00-5.23)	<0.001
*Women*	450	1.0	1.96 (0.94-4.10)	3.07 (1.50-6.28)	5.76 (2.84-11.69)	<0.001
*Adjusted*^b^	200	1.0	2.80 (0.69-11.40)	6.82 (1.50-31.08)	5.63 (1.50-21.11)	0.010
*Adjusted*^c^	183	1.0	3.99 (0.84-18.83)	9.13 (1.67-49.95)	7.09 (1.68-29.88)	0.010

Conditional logistic regression analysis with odds ratio and 95% confidence interval.

^a^P-Apo A1 quartile limits were for men, 1.27, 1.41, 1.56 and for women, 1.42, 1.60, 1.75 g/L. P-Apo B quartile limits were for men, 0.99, 1.17, 1.31 and for women, 0.96, 1.18 and 1.39 g/L. P-ApoB/Apo A1 ratio quartile limits were for men, 0.686, 0.817, 0.966 and for women, 0.601, 0.733, 0.890; ^b^Adjusted for smoking, systolic blood pressure, BMI and CRP; ^c^Adjusted for diabetes, smoking, systolic blood pressure, BMI and CRP.

### Determinants of Apo A1

The correlations of clinical data, analytes and MTHFR genotypes with Apo A1 in univariate linear regression analysis for men and women separately are seen in Table 3. tHcy in plasma was not associated with Apo A1 levels. Subset analysis of women defined as postmenopausal (age 56 years and older) showed the same significant associations as for all women. The same was seen for women below 56 years.

**Table 3:** Univariate linear regression versus Apo A1 for men and women separately

	**Men**			**Women**		
Variables	All	Cases	Referents	All	Cases	Referents
BMI, kg/m^2^	0.061 (<0.001)	0.018 (0.005)	0.078 (<0.001)	0.017 (0.022)	0.016	0.010
Age at inclusion, years	0.002	0.005	<0.001	0.030 (<0.001)	0.039 (0.008)	0.027 (0.003)
Diabetes, (yes/no)	0.005 (0.015)	0.005	<0.001	0.006	<0.001	0.035 (0.013)
Systolic BP, mm Hg	<0.001	0.003	<0.001	0.011	0.087 (0.004)	0.002
Current smoking, (yes/no)	0.001	<0.001	<0.001	0.024 (0.001)	<0.001	0.019 (0.010)
Homocysteine, μmol/L	<0.001	<0.001	<0.001	<0.001	<0.001	<0.001
Vitamin B12, pmol/L	0.011 (<0.001)	<0.001	0.018 (<0.001)	<0.001	<0.001	0.736
Folate, nmol/L	0.023 (<0.001)	0.003	0.026 (<0.001)	0.001	<0.001	<0.001
Creatinine, μmol/L	0.007 (0.005)	0.015 (0.012)	0.003	<0.001	<0.001	−0.004
Albumin, g/L	0.007 (0.004)	0.001	0.005 (0.040)	<0.001	<0.001	<0.001
Cystatin-C, mg/L	<0.001	0.010 (0.033)	<0.001	<0.001	0.010	<0.001
CRP log	0.041 (<0.001)	0.017 (0.006)	0.039 (<0.001)	0.067 (<0.001)	0.055 (0.002)	0.051 (<0.001)
*MTHFR 677C>T*	0.002	<0.001	0.004	<0.001	<0.001	0.001
*677 rec*^a^	0.002	<0.001	0.004 (0.048)	0.001	<0.001	0.002
*677 dom*^b^	0.002	<0.001	0.001	<0.001	0.003	<0.001
*MTHFR 1298A>C*	<0.001	<0.001	<0.001	<0.001	0.006	<0.001
*1298 rec*^c^	<0.001	<0.001	<0.001	<0.001	0.005	<0.001
*1298 dom*^d^	<0.001	<0.001	<0.001	<0.001	<0.001	<0.001

^a^MTHFR 677 rec: 0 = 677CC and CT, 1 = 677TT (recessive model); ^b^MTHFR 677 dom: 0 = 677CC, 1 = 677CT and TT (dominant model); ^c^MTHFR 1298 rec: 0 = 1298 AA and AC, 1 = 1298 CC (recessive model); ^d^MTHFR 1298 dom: 0 = 1298AA, 1 = 1298 AC and CC (dominant model).

R^2^ (p-value, if <0.05) is presented.

Among all men and male referents Vitamin B12 and folate levels were associated with Apo A1.

Univariate analyses were performed in subgroups based on fasting status. Only 3.6% (n=41) of the men had fasted for less than 4 hours. An association was seen between homocysteine and Apo A1 in this non-fasting group. But among the male fasting subjects no significant associations were seen for tHcy with Apo A1. In contrast, folate remained significantly associated with Apo A1 among men that had fasted more than 8 hours. Among women no significant association was seen for tHcy and Apo A1 in the subgroups. For folate and Apo A1 a significant association was seen in the fasting group of 4–8 hours.

Regarding MTHFR genotypes, no associations with apolipoproteins were seen in all men and women respectively, but among male referents, a negative association was found between Apo A1 and MTHFR 677 in a recessive model.

Multiple regression analysis for the association with ApoA1 is presented in Table 4 and for subgroups in Table 5. Among all men, Apo A1 was associated with BMI, age, systolic blood pressure, vitamin B12, folate, creatinine and CRP. The associations between Apo A1 and analytes were consistent in male referents, except for creatinine. For male cases Apo A1 were associated with creatinine and CRP. The association between Apo A1 and clinical data were consistent in male cases but only for BMI in male referents. Among all women and female referents, folate and CRP were associated with Apo A1. No associations were seen for female cases. Adding homocysteine to the multiple models did not change the results for men, but significance was lost for folate among women. Among all men, R^2^ of the total model was 0.144, i.e. it explains 14.4% of the variation in Apo A1 levels. Clinical data and plasma variables had R^2^ values 0.085 and 0.074 respectively. Among all women, corresponding R^2^ values were 0.106, 0.066 and 0.065.

**Table 4:** Multiple regression models on factors influencing Apo A1 and Apo B levels for all men and women separately

	**Apo A1 Men**			**Women**			**Apo B Men**			**Women**		
**R**^**2 **^**Complete Model**	**0.144**			**0.106**			**0.058**			**0.191**		
	**Beta**	**SE**	**p**	**Beta**	**SE**	**p**	**Beta**	**SE**	**p**	**Beta**	**SE**	**p**
Clinical data												
BMI	−0.014	0.002	**<0.001**	−0.003	0.004	0.450	0.013	0.003	**<0.001**	0.007	0.005	0.169
Age at inclusion	0.003	0.001	**0.004**	0.001	0.002	0.603	0.003	0.001	**0.031**	0.010	0.003	**0.001**
Diabetes	0.020	0.059	0.731	0.059	0.129	0.648	−0.056	0.078	0.471	0.165	0.163	0.314
Systolic BP	0.001	0.000	**0.024**	0.001	0.001	0.112	0.000	0.001	0.915	0.004	0.001	**0.001**
Current smoking	0.094	0.064	0.143	0.054	0.139	0.698	−0.005	0.085	0.957	0.428	0.176	**0.016**
Analytes												
Vitamin B12	0.000	0.000	**0.016**	0.000	0.000	0.476	0.000	0.000	0.401	0.000	0.000	0.810
Folate	0.005	0.001	**0.001**	0.006	0.003	**0.031**	−0.002	0.002	0.287	−0.002	0.003	0.486
Creatinine	−0.001	0.001	**0.013**	0.002	0.002	0.229	0.001	0.001	0.139	0.006	0.002	**0.011**
CRP log	−0.078	0.016	**<0.001**	−0.124	0.037	**0.001**	0.019	0.021	0.352	0.032	0.047	0.501
Albumin	0.003	0.002	0.110	−0.001	0.002	0.474	0.010	0.003	**<0.001**	0.000	0.003	0.885

P-values <0.05 are shown in bold.

**Table 5:** Multiple regression models on factors influencing Apo A1 levels in referents and cases, respectively separated in men and women

	**Referents Men**			**Women**			**Cases Men**			**Women**		
**R**^**2 **^**Complete model**	**0.140**			**0.099**			**0.112**			**0.078**		
	**Beta**	**SE**	**p**	**Beta**	**SE**	**p**	**Beta**	**SE**	**p**	**Beta**	**SE**	**p**
Clinical data												
BMI	−0.015	0.003	**<0.001**	0.002	0.005	0.739	−0.012	0.003	**0.001**	−0.006	0.006	0.311
Age at inclusion	0.002	0.001	0.108	0.001	0.003	0.664	0.004	0.002	**0.025**	0.004	0.004	0.334
Diabetes	0.042	0.077	0.584	−0.082	0.164	0.619	−0.039	0.092	0.675	−0.248	0.231	0.288
Systolic BP	0.001	0.001	0.171	0.001	0.001	0.266	0.002	0.001	**0.024**	0.001	0.001	0.344
Current smoking	0.097	0.087	0.262	0.069	0.177	0.699	0.054	0.098	0.582	−0.248	0.231	0.288
Analytes												
Vitamin B12	0.000	0.000	**0.017**	0.000	0.000	0.693	0.001	0.001	0.347	0.000	0.000	0.468
Folate	0.004	0.002	**0.009**	0.007	0.003	**0.030**	0.004	0.003	0.157	0.002	0.005	0.672
Creatinine	−0.001	0.001	0.338	0.000	0.002	0.941	−0.002	0.001	**0.008**	0.003	0.003	0.223
CRP log	−0.081	0.019	**<0.001**	−0.144	0.049	**0.004**	−0.067	0.028	**0.019**	−0.095	0.072	0.192
Albumin	0.002	0.003	0.357	0.000	0.002	0.845	0.005	0.004	0.213	−0.003	0.008	0.722

P-values <0.05 are shown in bold.

### Determinants of Apo B

The correlations of clinical data, analytes and MTHFR genotypes with Apo B in univariate linear regression analysis for men and women separately are seen in Table 6. For tHcy there was an association with Apo B levels among all women and female referents and among the same, folate was negatively associated with Apo B. tHcy and folate did not remain significant in a subset analyses of women defined as postmenopausal (age 56 years and older). Analyses in subgroups based on fasting status showed no significant associations for tHcy and Apo B among men. In women the significant association remained in the non-fasting group. For folate and Apo B no significant association was seen in the subgroups among men. In women significant associations remained in the non-fasting group and in the group that had fasted 4–8 hours.

**Table 6:** Univariate linear regression versus Apo B for men and women separately

	**Men**			**Women**		
Variables	All	Cases	Referents	All	Cases	Referents
BMI, kg/m^2^	0.028 (<0.001)	<0.001	0.046 (<0.001)	0.034 (0.002)	0.012	0.035 (0.009)
Age at inclusion, years	0.002	0.005	0.015 (<0.001)	0.062 (<0.001)	<0.001	0.115 (<0.001)
Diabetes, (yes/no)	<0.001	<0.001	<0.001	<0.001	<0.001	<0.001
Systolic BP, mm Hg	0.005 (0.010)	<0.001	0.012 (0.002)	0.070 (<0.001)	0.003	0.081 (<0.001)
Current smoking, (yes/no)	0.007 (0.004)	<0.001	<0.001	<0.001	<0.001	<0.001
Homocysteine, μmol/L	<0.001	0.006	<0.001	0.013 (0.009)	<0.001	0.021 (0.008)
Vitamin B12, pmol/L	<0.001	<0.001	<0.001	<0.001	<0.001	<0.001
Folate, nmol/L	0.001	<0.001	<0.001	0.012 (0.012)	<0.001	0.019 (0.012)
Creatinine, μmol/L	0.001	0.001	<0.001	0.009 (0.028)	<0.001	0.033 (0.001)
Albumin, g/L	0.005 (0.015)	0.022 (0.003)	0.003	<0.001	<0.001	<0.001
Cystatin-C, mg/L	<0.001	0.003	<0.001	0.007	<0.001	0.005
CRP log	0.067 (<0.001)	0.017 (0.006)	0.039 (<0.001)	0.067 (<0.001)	0.055 (0.002)	0.051 (<0.001)
*MTHFR 677C>T*	<0.001	<0.001	<0.001	0.004	0.004	<0.001
*677 rec*^a^	<0.001	<0.001	0.001	0.001	0.008	<0.001
*677 dom*^b^	<0.001	<0.001	<0.001	<0.001	<0.001	<0.001
*MTHFR 1298A>C*	0.001	0.002	0.008 (0.026)	<0.001	<0.001	<0.001
*1298 rec*^c^	0.001	0.003	0.009 (0.007)	0.002	0.005	<0.001
*1298 dom*^d^	<0.001	0.002	<0.001	<0.001	<0.001	<0.001

^a^MTHFR 677 rec: 0 = 677CC and CT, 1 = 677TT (recessive model); ^b^MTHFR 677 dom: 0 = 677CC, 1 = 677CT and TT (dominant model); ^c^MTHFR 1298 rec: 0 = 1298 AA and AC, 1 = 1298 CC (recessive model); ^d^MTHFR 1298 dom: 0 = 1298AA, 1 = 1298 AC and CC (dominant model).

R^2^ (p-value, if <0.05) is presented.

Regarding MTHFR genotypes, no associations with apolipoproteins were seen in all men and women respectively, but in male referents between Apo B and MTHFR 1298A>C and with 1298 in a recessive model. Multiple regression analysis for the association with Apo B is presented in Table 4 and for subgroups in Table 7. For Apo B an association was seen in all men for BMI, age and albumin. The same was seen for male referents but among male cases an association was seen only for albumin. For all women an association was seen with age, systolic blood pressure, smoking and creatinine. In female referents positive association was seen for creatinine and all clinical data, except BMI. Among female cases a positive correlation was seen for albumin only. In men R^2^ values were 0.058, 0.045 and 0.018 in the total model, for clinical data and for plasma variables respectively and the R^2^ values for women were 0.191, 0.170 and 0.022. Adding homocysteine to the model did not change the major findings, but in female referents the correlation between Apo B and diabetes was no longer significant and in male cases a significant correlation between Apo B and creatinine was seen.

**Table 7:** Multiple regression models on factors influencing Apo B levels in referents and cases, respectively separated in men and women

	**Referents Men**			**Women**			**Cases Men**			**Women**		
**R**^**2 **^**Complete model**	**0.068**			**0.283**			**0.030**			**0.131**		
	**Beta**	**SE**	**p**	**Beta**	**SE**	**p**	**Beta**	**SE**	**p**	**Beta**	**SE**	**p**
Clinical data												
BMI	0.015	0.003	**<0.001**	−0.001	0.006	0.926	0.005	0.005	0.323	0.010	0.008	0.171
Age at inclusion	0.004	0.001	**0.001**	0.015	0.004	**<0.001**	0.000	0.002	0.894	−0.003	0.005	0.535
Diabetes	−0.002	0.096	0.986	0.490	0.194	**0.013**	−0.071	0.127	0.576	−0.237	0.265	0.376
Systolic BP	0.000	0.001	0.814	0.004	0.002	**0.017**	−0.001	0.001	0.242	0.004	0.002	0.037
Current smoking	0.049	0.107	0.649	0.659	0.209	**0.002**	−0.029	0.134	0.830	0.029	0.283	0.918
Analytes												
Vitamin B12	0.000	0.000	0.151	0.000	0.000	0.903	0.000	0.000	0.422	0.000	0.000	0.910
Folate	−0.001	0.002	0.633	−0.002	0.004	0.594	−0.001	0.004	0.867	−0.001	0.006	0.927
Creatinine	0.000	0.001	0.853	0.008	0.003	**0.008**	0.002	0.001	0.057	0.002	0.003	0.491
CRP log	0.027	0.024	0.255	0.033	0.058	0.565	−0.034	0.039	0.384	0.024	0.088	0.785
Albumin	0.009	0.003	**0.005**	−0.001	0.003	0.621	0.016	0.005	**0.003**	0.031	0.010	**0.004**

P-values <0.05 are shown in bold.

## Discussion

This study on the relationship between apolipoproteins, B-vitamins, homocysteine and MTHFR is to our knowledge the largest, including both men and women. We have previously reported that homocysteine levels were positively associated and folate concentrations in plasma were inversely associated with first myocardial infarction in this cohort [1]. The associations were seen independently of each other and of a number of cardiovascular risk factors, including apolipoprotein levels. No clear risk relationship was apparent for plasma vitamin B12 concentrations, and vitamin B12, B6 and B2 intake.

As expected, Apo A1 and Apo B showed strong associations with risk for first myocardial infarction, except the association with Apo A1 in women where the odds ratio increased after adjustments and was no longer significant. Adjusting Apo A1 for folate did not change the significant findings, which is in line with our previously reported study [1].

Analyzing over 1500 subjects, from a population without folate fortification, we found no clear relationship between plasma tHcy and Apo A1 levels in multiple regression. In contrast folate was positively associated with Apo A1 levels in multiple analyses in all men and women, as well as among male and female referents. This association was not seen among cases which could be due to lower statistical power as the beta-values for male cases and referents are similar (Table 5). It is also possible that something else interferes in the subjects that later develop MI. Vitamin B12 showed an association with Apo A1 in men, i.e. among male referents.

The MTHFR677C>T polymorphism, a major genetic determinant of tHcy, showed no association with Apo A1 levels, with the exception of male referents where an association was seen for Apo A1 using a recessive model. The significance of this is unclear. In the multiple models, the plasma variables and the clinical data had approximately the same explanatory power on the variation in Apo A1.

For Apo B levels no association was seen for B-vitamins in multiple regression models. tHcy had a positive association in univariate regression analysis with Apo B among all women and female referents. Apo B variation among women was explained to a higher degree by clinical data compared to Apo A1.

Previous studies [20,22-25] have reported that increased plasma tHcy is associated with decreased Apo A1 levels. Two early studies reported a negative correlation in men with coronary artery disease [23,27] with 40 and 60 subjects respectively. In a Finnish cohort of about the same size as ours, Apo A1 was associated with tHcy in a multivariate regression model [22]. Compared to our cohort their subjects were much younger (24–39 years). They reported no association between MTHFR and Apo A1 levels which is in agreement with our results. None of the studies mentioned above included analysis of the major dietary determinants of tHcy, i.e. plasma folate and vitamin B12. A Norwegian case–control study showed that whole blood folate correlated positively and tHcy negatively with Apo A1 and HDL when merging cases and controls (n=210), but no further statistical analysis of these associations was performed [34]. A large study (middle aged men, n=2775) with multiple biomarkers linked to coronary heart disease risk, failed to show an association between plasma levels of Apo A1 or HDL and plasma levels of tHcy or folate [31]. They further analyzed associations with 948 SNPs in 122 candidate genes. SNPs in the APOA5-A4-C3-A1 gene were associated with triglycerides, HDL, Apo A1, tHcy and folate. After adjustment for multiple testing, folate but not homocysteine, was still significantly associated with that gene. They found no association between MTHFR and lipids.

A study on heterozygous familial hypercholesterolemia reported that higher plasma homocysteine levels and MTHFR 677 TT genotype were associated with lower HDL plasma values [21]. Consistent with this, results from a cross-sectional study in a large population, who had undergone a diagnostic coronary angiography, show that plasma homocysteine, but not cysteine, correlated negatively with Apo A1 [25]. Similar findings were reported in a study with elderly volunteers (n=667, aged 60–85 years) with peripheral arterial disease [20]. They reported a negative correlation for homocysteine with Apo A1 as well as with HDL, but found no association for folate. Vitamin B12 was positively, and methylmalonic acid negatively associated with Apo A1 in univariate analysis only. The results of our study do not support these reported findings. One reason could be that in contrast to the others studies, the participants in our study were free from previous history of MI and stroke. Concurrent disease may influence the results. This can be exemplified by a study with type 2 diabetic patients treated with fenofibrate, were baseline HDL and Apo A1 correlated inversely with homocysteine in both fenofibrate and placebo groups before treatment [36].

One study in cystathionine β-synthase (Cbs)-deficient mice suggested that cysteinemia rather than homocysteinemia is negatively correlated with Apo A1 [37]. Another study on humans showed a positive correlation between total cysteine and total cholesterol, and Apo B [30]. They also reported that taurin was inversely related to HDL cholesterol and Apo A1. No association with homocysteine was found.

Folate, in the form of 5-methyl tetrahydrofolate, is the main source of methyl groups used for methylation, for example by DNA methyl transferases. Thus, one-carbon metabolism is closely linked to methylation of genes, for example promotor regions. This epigenetic mechanism may either silence or activate a gene. We have previously published data on the effects on CpG island methylator phenotypes [38]. Altered DNA methylation patterns in atherosclerosis, both hyper- and hypomethylation, have been suggested to be secondary to a decrease in S-adenosyl methionine, the main methyl group donor which is highly dependent on the availability of folate and vitamin B12 [39]. One possible link between one-carbon metabolism and lipid metabolism is folate-dependent epigenetic effects in genes, such as the modulation of peroxisome proliferator-activated receptor (PPAR) α in mouse embryos [40,41]. PPAR α agonists such as fibrates have been shown to upregulate transcription of the human Apo A1 gene but the mechanism in humans is not known [42,43].

Other studies in mice and cells including the modulation of PPAR α, supports a negative correlation between tHcy and Apo A1 [27,44]. Also, hyperhomocysteinemia may inhibit HDL biosynthesis and function by reduced hepatic Apo A1 protein expression, and increased HDL-C clearance [24].

A main strength in our study is the design where we could study subjects that did not have previous MI, stroke or cancer, at inclusion, with both men and women in the cohort. These findings are probably representative for the general population as we only excluded subjects with prior MI, stroke or cancer and we were able to analyze separately those who later did or did not have a first MI. We also excluded all subjects with lipid lowering drugs. Other strengths are that we analyzed the B-vitamins folate and B12, and that the results were not confounded by folate fortification. A limitation of this study is that lifestyle factors can be a confounding factor, as healthy eating and exercise could lead to higher levels of both Apo A1 and folate. The number of cases may be too low to reveal a weak association between folate and Apo A1. Not all subjects were fasting at time of blood sampling. This may have had an effect on the results, as tHcy may be influenced by a high methionine meal. However, the number of subjects that were not fasting was low and excluding those subjects had no effect on the associations. As alcohol is known to have impact on lipoproteins, it is also a limitation that alcohol intake data was only available for a subset and thus could not be corrected for [45].

## Conclusions

In conclusion, tHcy was not associated with Apo A1 levels in a large cohort with over 1500 subjects without folate fortification. Instead, plasma folate, the major dietary determinant of tHcy, was found to be independently and positively associated with Apo A1 levels. This association was seen for both men and women, but restricted to referents. Those who later developed MI showed no association with folate. Future studies on the association between tHcy and endpoints or biomarkers should also take into account major determinants of tHcy, e.g. folate.

## Methods

### Study population

This was a prospective incident nested case-referent study within the Northern Sweden Health and Disease Study Cohort (NSHDSC) [46]. The cohort has been collected using populations-based methodology: The Northern Sweden WHO Monitoring of Trends and determinants in Cardiovascular Disease (MONICA) study, the Västerbotten Intervention Project (VIP) and the local Mammography Screening Project (MSP). MONICA randomly selected and invited 25–74 year old subjects who participated in four health surveys between 1986 and 1999. The mean participation rate was about 77% [47]. VIP which was initiated in 1985, invited all subjects to their local primary health care center the year they turned 30, 40, 50, 60 years (since 1996 at 40, 50 and 60 years of age). The design of VIP was similar to the MONICA population surveys. Mean participation rate was approximately 60% and comparisons of social characteristics between participants and non-participants have shown little evidence of selection bias [48], implying the participants were representative of the population. In the local Mammography Screening Project(MSP), which was founded in 1995, all women approximately between 50–70 years were invited to undergo mammography, and at the same time they were invited to donate a blood sample to the Northern Sweden Medical Biobank [46]. Mean participation in mammography screening has been 85% and participation in screening and donation of blood sample has been 33%. There has been no mandatory folate fortification in Sweden.

All participants were invited to complete a standardized questionnaire including life-style factors and subjects were interviewed by a trained nurse for all sorts of medication used. Those participating within the MSP completed a short questionnaire. The standardized questionnaires were used to calculate alcohol intake (g/day). This information was only available for a subset (170 male and 35 female cases and 367 male and 78 female referents) and thus not included in the analysis.

In the study, all cases with fatal and non-fatal first MI and suspected fatal MI (ICD-9 410, ICD-10 I21-3), between the ages of 25 and 64 years and from 1 January 1985 to 31 December 1999 were included. The case identification was done in a standardized manner in accordance with WHO and MONICA criteria [47]. Using Swedish personal identity numbers as the linkage variable, hospital records, general practitioner´s reports, death certificates, and autopsy reports were screened. For cases, exclusion criteria were, previous MI, stroke or cancer diagnosis within 5years prior to and 1 year after MI. Two matching referents for each case were randomly selected. Referents were excluded for MI, stroke, cancer or death prior to the time of diagnosis of the index case.

Diabetes was defined as fasting plasma glucose ≥ 7.0 mmol/L and/or post-load plasma glucose ≥ 11.0 mmol/L (12.2 mmol/L in VIP, in which capillary plasma was used) and/or self-reported diabetes. For classification as non-diabetic, measurement of fasting or post-load glucose was required. Fasting glucose and/or 2-hour oral glucose load tests were available for 444 cases and 855 referents. Patients with lipid-lowering drugs (n=9) were excluded. Hypertension was defined as systolic blood pressure (BP) ≥ 140 mm Hg and/or diastolic blood pressure ≥ 90 mm Hg and/or use of medication to lower blood pressure. Current smoking was defined as daily smoking, excluding non-, previous-, and non-daily smokers. Information about pre- or postmenopausal status among women was not available in this study. Postmenopausal woman was therefore defined as ≥ 56 years of age at inclusion (n=311), the age when 95% are estimated to be in menopausal status in a Swedish material [49]. According to this study females below the age of 45 years would include a few percent postmenopausal women. This group was small (n=24) in our study.

All participants have given written consent. The Research Ethics Committee of Umeå University, Umeå and the National Computer Data Inspection Board have approved the data handling procedures.

### Blood sampling and laboratory procedures

Peripheral venous blood samples were collected in evacuated test tubes containing heparin, in MONICA and VIP, generally in the morning after at least four hours fasting. In MSP blood samples were collected throughout the day. In the study, 66.9% (n=762) of the men had fasted for more than 8 hours and 15.7% (n=179) had fasted between 4–8 hours, while 3.6% (n=41) had fasted for less than 4 hours. Information about fasting status was missing for 13.8% (n=157) of the men. Corresponding fasting status for women was, 36.5% (n=168), 14.8% (n=68) and 43.0% (n=198). Information about fasting status was missing for 5.7% (n=26) of the women.

Plasma was aliquoted after centrifugation at 1500g for 15 minutes. Until analysis they were stored frozen at −80˚C. Plasma specimens were analyzed in triplets of one case and two referents. To avoid systemic bias and interassay variability the position for the case was varied in random and the laboratory staff were blinded to case and referent status.

Apo A1 and Apo B were analyzed with immune turbidimetry on a Hitachi 911 multianalyzer with reagents from DAKO (Copenhagen, Denmark). Homocysteine was analyzed in batch with fluorescence polarization immunoassay on an IMx® unit (Abbott Laboratories, IL, USA). P-B12 and P-folate were analyzed in batch using DPC® Solid Phase No Boil Dualcount radio assay (Diagnostic Products Corporation, CA, USA). Creatinine, albumin and cystatin C were analyzed with a Hitachi 911 multianalyzer, creatinine (Crea plus, enzymatic method) with kits from Roche/Boehringer (Mannheim, Germany). Albumin was analyzed with immune turbidimetry and Cystatin C with an immunological method, both with reagents from DAKO (Copenhagen, Denmark). C-reactive protein (CRP) was analyzed with an automated method, IMMULITE 1 (Diagnostic Products Corporation, Los Angeles, CA, USA). Analyses below were analyzed in batch and thus intra-assay coefficients of variation are presented. For Apo A1 coefficients of variation (CV:s) were 0.96% and 0.61% at concentrations 0,91 and 1,65 g/L, respectively and for Apo B 1.21% and 0.62% at concentrations 0.47 and 0.81 g/L, respectively. Homocysteine had CV:s of 2.7% and 1.9% at concentrations 13.1 μmol/L and 25.7 μmol/L respectively. P-folate had CV:s of 15.8% and 12.1% at concentrations 7.3 mol/L and 30.4 nmol/L, respectively and P-B12 16.5% and 10.4% at the concentrations of 263 pmol/L and 1001 pmol/L. For control samples, for creatinine CV:s were 1.59% and 3.21% at concentrations 84 and 367 μmol/L respectively, for albumin 0.89% and 2.25% at 38.0 g/L and 64.3 g/L and for Cystatin C 2.90% and 1.20% at 1.12 and 4.59 mg/L. For CRP the interassay coefficient of variation was <6%.

MTHFR genotyping was performed by the TaqMan allelic discrimination method, using Minor Groove Binder (MGB) probes. TaqMan assays and reagents were from Applied Biosystems (Foster City, CA, USA). The PCR reactions were performed on GeneAmp PCR system 9700, PCR programs (Assay-on- Demand, ABI) at the Center for Genome Research, Umeå University and the PCR products were analyzed on the ABI PRISM 7900HT Sequence Detection System.

### Statistical analysis

Calculations were performed using IBM SPSS Statistics version 19.0 (IBM Corporation, New York, NY, USA). Hardy-Weinberg equilibrium was calculated, based on the χ^2^-test.

To compare baseline characteristics and study variables for cases and referents T-test and Chi-square test for independence were used for continues and categorical variables respectively. Plasma apolipoprotein quartile limits, based on referents, were separately calculated for men and women and treated as categorical variables, except when testing for trend, where they were included as continuous linear variables in the regression analyses. Odds ratios (OR) for disease and 95% confidence intervals (CI) were calculated by conditional logistic regression. Adjustment was done only for folate and with two other models both including smoking, systolic blood pressure, BMI and CRP but with and without diabetes. Missing values were treated as missing.

To find factors influencing Apo A1, linear regression analysis was performed. Normal distribution could not be applied for CRP in our population and instead transformation was done to logarithmic values. In univariate linear regression MTHFR genotypes were coded as follows: one model using the three outcomes coded 0,1,2; one recessive model (wild type and heterozygote mutant v.s. homozygote mutant ) the other, dominant model (wild type v.s. heterozygote mutant and homozygote mutant).

Analytes and genotypes that were non-significantly (p<0.05) associated with apolipoproteins in univariate analysis of variance were excluded in the multiple regression models. An exception was homocysteine were we wanted to test the hypothesis regardless the result in univariate analysis.

## Abbreviations

tHcy: Total plasma homocysteine; Apo A1: Apolipoprotein A1; Apo B: Apolipoprotein B; MTHFR: Methylenetetrahydrofolate reductase; MI: Myocardial infarction.

## Competing interests

The authors declare that they have no competing interests.

## Authors' contributions

JHJ, GH, LW and ME designed the cohorts. The study was designed by JH, ES, ME, JHJ and OJ. JH carried out biochemical and genetic analysis. JH and ES performed the statistical analysis and interpreted the data. Drafting was made by JH and ES and all other authors revised it critically. All gave final approval of the version to be published.
